# The Utilization of Gleason Grade as the Primary Criterion for Ordering Nuclear Bone Scan in Newly Diagnosed Prostate Cancer Patients

**DOI:** 10.1100/tsw.2009.113

**Published:** 2009-10-02

**Authors:** Chad W. M. Ritenour, John T. Abbott, Michael Goodman, Naomi Alazraki, Fray F. Marshall, Muta M. Issa

**Affiliations:** ^1^ Department of Urology, Emory University, School of Medicine, Atlanta, GA, United States; ^2^ Department of Urology, Veterans Affairs Medical Center, Atlanta, GA, USA; ^3^ Deparment of Epidemiology, Rollins School of Public Health, Emory University, Atlanta, GA, United States; ^4^ Division of Nuclear Medicine, Veterans Affairs Medical Center, Atlanta, GA, USA

**Keywords:** prostate, prostatic neoplasm, bone scan, staging, Gleason grade, prostatespecific antigen

## Abstract

Utilization of nuclear bone scans for staging newly diagnosed prostate cancer has decreased dramatically due to PSA-driven stage migration. The current criteria for performing bone scans are based on limited historical data. This study evaluates serum PSA and Gleason grade in predicting positive scans in a contemporary large series of newly diagnosed prostate cancer patients. Eight hundred consecutive cases of newly diagnosed prostate cancer over a 64-month period underwent a staging nuclear scan. All subjects had histologically confirmed cancer. The relationship between PSA, Gleason grade, and bone scan was examined by calculating series of crude, stratified, and adjusted odds ratios with corresponding 95% confidence intervals. Four percent (32/800) of all bone scans were positive. This proportion was significantly lower in patients with Gleason score ≤7 (1.9%) vs. Gleason score ≥8 (18.8%, *p* < 0.001). Among patients with Gleason score ≤7, the rate of positive bones scans was 70-fold higher when the PSA was >30 ng/ml compared to ≤30 ng/ml (*p* < 0.001). For Gleason score ≥8, the rate was significantly higher (27.9 vs. 0%) when PSA was >10 ng/ml compared to ≤10 ng/ml (*p* = 0.002). The combination of Gleason score and PSA enhances predictability of bone scans in newly diagnosed prostate cancer patients. The PSA threshold for ordering bone scans should be adjusted according to Gleason score. For patients with Gleason scores ≤7, we recommend a bone scan if the PSA is >30 ng/ml. However, for patients with a high Gleason score (8–10), we recommend a bone scan if the PSA is >10 ng/ml.

## INTRODUCTION

Prostate cancer is the most common internal cancer and the second leading cause of cancer death in American men. The American Cancer Society estimates that approximately 186,320 new cases of prostate cancer were diagnosed in 2008 in the U.S. and 28,660 men died of the disease. Current recommendations of annual screening with the prostate specific antigen test (PSA) and digital rectal examination (DRE) have led to a stage migration with a shift toward clinically localized disease[1]. Ninety percent of all prostate cancers are discovered in the local and regional stages with a 5-year survival approaching 100%[2]. The relative 10- and 15-year survival is 91 and 76%, respectively. Recommendation of the appropriate treatment plan requires accurate staging of the disease. At this time, there is no formal consensus on the pretreatment staging for prostate cancer[3,4].

Nuclear bone scan is the current gold standard for staging bone metastases compared to plain skeletal radiography or serum alkaline phosphatase and acid phosphatase levels[5,6]. Until a decade ago, bone scans were routinely performed on newly diagnosed prostate cancer patients[3]. With the introduction of PSA testing, such nondiscriminatory use of bone scans was reduced. Various recommendations were presented to allow more selectivity in performing bone scans. Subgroups of prostate cancer patients with minimal risk for bone metastases were excluded in an attempt to minimize unnecessary bone scans and decrease costs. The American Urological Association (AUA) recommendation states that a bone scan is not indicated in an asymptomatic patient with localized disease and a PSA of ≤20 ng/ml[7]. The American College of Radiology (ACR), on the other hand, feels that bone scans are necessary unless the PSA is <10 ng/ml[8]. Both the AUA and ACR suggest the consideration of bone scans in higher-risk cases including advanced-grade and -stage cancer[7,8]. Such information points to current discrepancy in the PSA threshold, as well as a lack of clarification and specifics on the high-risk subgroup. Further, these recommendations were based on dated series that did not account for the current variation in disease presentation, staging, and demographics. The utilization of bone scans seems to have dropped prematurely irrespective of these unresolved issues[4]. Patients with lowest risk continue to undergo unnecessary testing, while those at highest risk may not be appropriately evaluated.

While PSA and Gleason grade have been utilized as prognostic factors to predict staging such as lymph node metastases[9,10], their combined role and value in predicting the results of bone scans have not been specifically studied. The low utilization of bone scans adds further challenges to the evaluation of the impact these parameters. In a large institution like the Veterans Affairs (VA) Health Care System (7.84 million veteran enrollees)[11], over 95% of patients are men and the majority of this male population is ≥50 years of age. These men are routinely screened for prostate cancer, and many are diagnosed with the disease and undergo subsequent staging. Bone scans are performed routinely on all newly diagnosed prostate cancer patients in our institution. We undertook a retrospective analysis of this contemporary consecutive series of newly diagnosed prostate cancer patients that have undergone staging with bone scans.

## METHODS AND MATERIALS

The study was approved by the Research and Development Board at the Atlanta VA Medical Center and the Institutional Review Board at Emory University. This study is a retrospective analysis of a series of consecutive veterans with newly diagnosed prostate cancer at the Atlanta VA Medical Center over a 64-month period between November 2000 and March 2006. Patients were referred to urology by their primary care providers for evaluation of abnormal DRE, elevated serum PSA, or both. As such, the indications for prostate biopsy included these criteria. While there was no strict limit regarding age, patients with advanced age and/or significant comorbidities were counseled regarding the risks and benefits of undergoing prostate biopsy.

Standard urologic history and physical examination including DRE was performed prior to the biopsy. Transrectal ultrasound was performed under local anesthesia using Logiq™200 Pro Series System

(General Electric). Standard prostate biopsy was performed under ultrasound guidance using an 18-gauge needle with a Pro-Mag I 2.2 MD Tech spring-loaded biopsy device. The biopsy cores were obtained from the apex, mid region, and base of the peripheral zone of each prostate lobe. When indicated, particularly in substantially enlarged prostates, additional biopsy cores were obtained from the lateral aspect of the peripheral zone and from the transitional zone. The number of biopsy cores ranged between eight and 16. Patients were enrolled in the study when the diagnosis of prostate cancer was confirmed on histopathology. The number of positive biopsy cores and the percentage volume of cancer were not inclusion criteria. All biopsy specimens were reviewed by experienced pathologists in accordance with the standard Gleason grading criteria.

Eight hundred (800) consecutive cases of prostate cancer diagnosed during the 64-month period were included in the analysis. All patients underwent nuclear bone scan for staging. (Bone scan was performed using 20 mCi Tc 99mMDP [methylene diphosphonate] injected intravenously and followed at 2–3 h with planar imaging supplemented with SPECT as needed for abnormalities that were questionable or of uncertain location on planar images.) The relationship between the serum PSA, Gleason grade, and the result of the bone scan was examined by calculating series of crude, stratified, and adjusted odd ratios (OR) with corresponding 95% confidence intervals (95% CI). The reference level for tests of statistical significance was based on a $p$ value of 0.05. The association between the exposure and outcome was considered statistically significant when the $p$ value was $\leq 0.05$. All analyses were performed using SPSS 11.5 for Windows (LEAD Technologies, Chicago, IL) and SAS 8.02 for Windows (SAS Institute, Cary, NC) statistical software packages.

## RESULTS

Eight hundred (800) consecutive newly diagnosed prostate cancer patients were included in the analysis. Mean and median patient ages were 65 and 64 years, respectively. The results of 23.7% (190/800) of bone scans were equivocal/inconclusive necessitating additional radiological imaging for confirmation.

The overall proportion of positive bone scans for metastases in the series was 4% (32/800). None of the 546 patients with PSA $\leq 10$ ng/ml had positive bone scans compared to 12.6% (32/254) of positive bone scans in patients with PSA >10 ng/ml ($p < 0.001$). The overall proportion of positive bone scans was significantly higher in patients with Gleason score $\geq 8$ compared to those with Gleason score $\leq 7$ (18.8 vs. 1.9%; $p < 0.001$; OR 12.2, 95% CI: 5.5–27.4) (Fig. 1).

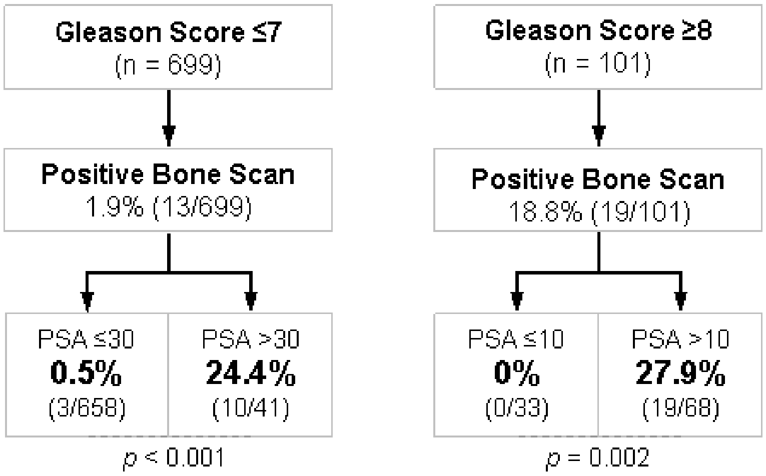

**FIGURE 1.** Results of bone scans based on Gleason score.

When the data were analyzed using Gleason grade as the primary threshold, the PSA cutoff value for positive bone scans changed. In patients with Gleason grade ≤7, the rate of positive bone scans was significantly higher (greater than 70-fold increase) with a PSA >30 ng/ml compared to a PSA ≤30 ng/ml (OR = 70.4; 95% CI: 16.7–342.1). There were only three patients with Gleason grade ≤7 and PSA ≤30 ng/ml who had positive scans. All three patients had PSA between 10 and 30 ng/ml (10.4, 12.3, and 13.1 ng/ml). There were no positive bone scans with PSA ≤10 ng/ml. Among patients with Gleason score ≥8, a PSA ≥10 ng/ml was associated with a 24% chance of a positive scan, compared to 0% if the PSA was less than 10 ng/ml (Fig. 1).

## DISCUSSION

The pretreatment staging of prostate cancer remains unclear and controversial, yet it plays a key role in management planning. Historically (pre-PSA era), nuclear scintigraphy was overutilized in the evaluation of bone metastases. That overusage was based on autopsy reports that as many as 80–85% of prostate cancer patients had bone metastases at death[12]. Not uncommonly, bone scan results are inconclusive and necessitate further investigations that, in turn, create anxiety for patients, add considerable cost, and delay therapy. Although the bone scan is a highly sensitive (90%) test to detect bone metastases[13], it is not specific and, particularly, solitary bone lesions may require other complementary imaging or biopsy. Statistically, a solitary bone lesion in a patient with known primary cancer is a metastasis in 30–50% of cases[14]. In our series, 23.7% (190/800) of bone scans were equivocal or inconclusive, and required additional radiological imaging, including plain films, computed tomography scanning, and magnetic resonance imaging, for confirmation.

Following the introduction of PSA, the test soon became the most commonly utilized prognostic parameter in prostate cancer[9]. In predicting bone metastases, a PSA threshold of >10 ng/ml is often currently used to trigger a bone scan in an asymptomatic patient[13]. Gleason grade, although used commonly in staging algorithms as a predictor of capsular penetration, seminal vesicle invasion, and lymph node metastases[10,14,15], has not been utilized to the same extent for predicting bone metastases[16,17]. Few investigators have observed positive correlation between histopathological grade and metastases on bone scan[9,18]. However, the disease stage was not representative of the stage migration currently seen in our prostate cancer population. Further, the histological grade was not standardized to one grading system. More recent studies demonstrated Gleason grade to be an independent predictor for positive bone scan and that its utilization may avoid a considerable number of bone scans[19,20]. These reports confirmed that routine bone scanning should not be mandatory in all cases of newly diagnosed prostate cancer.

Three patients with Gleason grade ≤7 and PSA ≤30 ng/ml had positive scans. All three patients had PSA between 10 and 30 ng/ml. In the first patient, treated with radiation, small early metastases had progressed minimally by the time of his death due to unrelated causes. In the second, treated with chemotherapy, his metastatic lesions have been stable over the past 4 years. The third patient died of a stroke within 4 months of his diagnosis and progression cannot be assessed.

Our data represent a modern sampling of newly diagnosed prostate cancer patients. The overall 4% rate of positive bone scans in our series is in line with ACS statistics[2]. All consecutive patients diagnosed with prostate cancer over a period of time received bone scans in an effort to avoid selection bias. The study is also unique because it evaluated both Gleason grade and PSA with equal emphasis. Considering the retrospective design of the study, further prospective investigations may be useful to confirm the results.

We concluded that a combination of Gleason score and PSA can be used more effectively than PSA alone in predicting the likelihood of positive bone scans in newly diagnosed prostate cancer patients. In particular, the predictability is enhanced when Gleason grade is used as a primary variable followed by the PSA as a secondary variable. For patients with Gleason score ≤7, the likelihood of a positive scan is increased only when the PSA is ≥30. For patients with Gleason score ≥8, the likelihood of a positive scan is high only when the PSA is ≥10.

By implementing this algorithm, bone scans become unnecessary in asymptomatic patients with PSA ≤10 ng/ml irrespective of the Gleason grade. Bone scans are equally unnecessary in patients with Gleason score ≤7 with PSA ≤30 ng/ml.

## CONCLUSIONS

The combination of Gleason score and PSA can be used to enhance the predictability of positive bone scans in newly diagnosed patients with prostate cancer. The PSA threshold value for performing bone scans is different depending on the Gleason score. When Gleason score is used as the primary variable, bone scans should be reserved for patients with Gleason score ≤7 when PSA ≥30 ng/ml, and for patients with Gleason grade ≥8 when PSA ≥10 ng/ml. Such practice can reduce unnecessary bone scans without compromising the staging accuracy. Nevertheless, discretion is always warranted when using such algorithms as there may be a few individual patients (e.g., those with a large tertiary component of pathologic Gleason score 5) who may be at higher risk for bony disease.

## ACKNOWLEDGMENT

The authors would like to thank Mihir Kamdar, M.D. and John Hall, PA-C for their assistance in data collection and analysis during the preparation of this project and manuscript.
